# Mental health and lifestyle health behaviors among commuter college students

**DOI:** 10.1080/28324765.2024.2436432

**Published:** 2024-12-07

**Authors:** Angela Marinilli Pinto, Andrea Bazzoli, Johanny Mercedes

**Affiliations:** a Psychology Department, Baruch College, City University of New York (CUNY), New York, USA; b Psychology Department, Baruch College, CUNY and the CUNY Graduate Center, New York, USA

**Keywords:** mental health, sleep, physical activity, anxiety, depression, commuter college

## Abstract

Commuter college students face unique circumstances and stressors that may impact their well-being, yet this is an area of research that has been largely unexplored. This study examines mental health and lifestyle health behaviors of commuter college students. Participants were 576 undergraduates (62% female, 81% non-White, 20.31 ± 3.02 years) attending an urban public commuter college. Participants completed a cross-sectional, anonymous, web-based survey that included validated measures of mood, anxiety, stress, sleep quality, and physical activity. Overall, 58.6% screened positive for depression, 37.8% screened positive for generalized anxiety, 57.5% had poor sleep quality, and only 23.3% met physical activity recommendations. More females screened positive for depression and anxiety and had poor sleep quality than males, and fewer females met activity recommendations. Being physically active and having good sleep quality were associated with better mental health. Findings reflect high rates of mental health problems and low engagement in health behaviors, particularly among women. Opportunities to address the well-being of commuter college students are discussed.

The well-being of college students in the US is a significant public health issue. According to the American College Health Association’s (American College Health Association [ACHA], 2024) survey, 35% of undergraduates have a lifetime diagnosis of an anxiety disorder, 27% have a lifetime diagnosis of depression, and 21% report feeling significant psychological distress (ACHA, 2024). These findings are also reflected in the 2023 annual report from the Center for Collegiate Mental Health (Center for Collegiate Mental Health [CCMH], (2024) which summarizes clinician assessments from 195 US colleges and universities and identifies anxiety, depression, and stress among the top concerns of students presenting to college counseling centers. In addition to the mental health burden, college students report getting insufficient sleep and physical activity- two lifestyle behaviors that impact both physical and mental health. One third of college students sleep less than 7 hours per night and more than 60% meet criteria for poor sleep quality (Becker et al., 2018). Additionally, less than half of college students meet recommended guidelines for physical activity which include at least 150 minutes per week of moderate-intensity aerobic activity and two days per week of moderate-intensity muscle strengthening activity (ACHA, 2024; US Department of Health and Human Services, 2018). Taken together, these findings raise concern for student well-being and educational success since mental health problems, poor sleep quality, and inactivity are associated with poorer academic outcomes (BlackDeer et al., 2023; Gilbert & Weaver, 2010; Pritchard & Wilson, 2003; Trockel et al., 2000).

Much of the evidence base for mental health and health behaviors of college students comes from national datasets and studies conducted in residential college settings. Little research has examined these factors among commuter students, yet commuters comprise the majority of US college students—about 84% according to data from the National Center for Education Statistics (Klechen, 2018). Moreover, commuter students are a heterogeneous group and even less research has evaluated whether well-being differs among commuter subgroups. Commuters include students attending residential colleges but living off-campus as well as students attending commuter schools, which are approximately 32% of US 4-year colleges (Weissberg & Owen, 2005). Theyinclude typical-aged full-time college students living at home or independently and older students with spouses and/or children who go to school while working full-time (Jacoby & Garland, 2004). Commuters may live near or far from campus and commute by car, public transportation, biking or walking (Jacoby, 1989; Kuh et al., 2001). While the specific circumstances of students’ commutes vary, the fact that they commute distinguishes their college experience from their residential college peers. For them, campus is a place they go to, not the place they live; thus they continue to interact with and balance demands in many other areas of their lives (Jacoby, 2000).

Available research shows that commuter students experience unique sources of stress that impact their well-being. They are more likely to work off campus, work more hours, and work to pay bills and tuition than residential students (Alfano & Eduljee, 2013; Burlison, 2015). Moreover, commuter students report more stress related to work, money, and time constraints than residential students (Forbus et al., 2010). Commuter students are also more likely to be managing multiple life roles while in college (Forbus et al., 2010; Newbold, 2015; Samuels et al., 2000). In addition to work responsibilities, students living at home or with families of their own are balancing academic demands with family obligations such as caring for children, siblings, or parents (Jacoby, 2000). Being split across these life domains strains not only their time and attention but also their mental and emotional resources (Newbold, 2015). Commuter students are more likely to be racial or ethnic minorities, have lower family incomes and be first generation college students (Gianoutsos & Rosser, 2014; Jacoby, 2000), factors which have been associated with increased psychological distress (Parker et al., 2023; Stebleton et al., 2014). First generation students also face the challenge of navigating college without the benefit of parents who can relate from their own experience. These students report lower perceived parental and family support (Jenkins et al., 2013; Sy et al., 2011) which may be magnified for first generation students living at home as they negotiate between family and academic expectations. Finally, the act of commuting itself is a stressor. Transportation difficulties such as cost, parking availability, reliability of public transit, and time spent traveling are realities for commuter students (Hernandez, 2002; Newbold, 2015; Schuette, 2023). The inconveniences of traveling to campus contribute to commuter student stress and reduced engagement, especially for students who live far away and schedule their time on campus around classes with off-campus days for work and other obligations. Analyzing data from the National Survey of Student Engagement, Kuh et al. (2001) showed that commuters who live driving distance from campus report less interaction with faculty, less involvement in enrichment opportunities outside the classroom (e.g., community service, internships, study abroad), and lower perceived support from the campus environment than residential students. Alfano and Eduljee (2013) also found that commuter students were less involved in school-sponsored social activities and less likely to feel part of the college community compared to students living on campus. Research by Kirk and Lewis (2015) identified time, transportation, and scheduling difficulties as specific barriers faced by commuter students in developing a sense of community and further showed that a lower sense of community was related to poorer psychological well-being.

Unlike college students who reside on campus, commuter students balance many competing life demands in addition to academics and often negotiate their college experience while feeling disconnected from the campus community. These circumstances present significant stressors that can be viewed from the perspective of resource scarcity theory, which posits that there are finite resources like time, energy, and money to meet needs and wants (Butler, 2007). When demands across different roles (e.g., school, personal life, work) exceed resources, this leads to increased stress and inter-role conflict which are related to reduced mental health and well-being (Giancola et al., 2009). Many commuter students manage these demands with limited actual or perceived social support, which can exacerbate mental health strain (Dennis et al., 2005; Parker et al., 2023). Simultaneously, the act of commuting occupies time that students could spend engaged in healthy lifestyle behaviors, such as physical activity and sleep, that mitigate the effect of stress and promote well-being (Ramar et al., 2021; Ridner et al., 2016; US Department of Health and Human Services, 2018). While these behaviors decline among college students generally (Buboltz et al., 2009; Keating et al., 2005; Small et al., 2013), commuter students may be particularly vulnerable because of their added time and role strains. Indeed, research shows that commuting is related to reduced physical activity and disturbed sleep (Christian, 2012; Hoehner et al., 2012; Raza et al., 2021). Moreover, physical activity and sleep are the primary health behaviors from which time is reallocated to accommodate commuting demands (Christian, 2012).

There is evidence from the general college literature that well-being differs between male and female college students, though results have been mixed. For example, Seehuus et al. (2021) reported greater mental health burden among women compared to men in a large sample of students from two small liberal arts colleges and Glavin et al. (2022) found higher rates of depression and anxiety among undergraduate females compared to males. In contrast, data from a large, multi-university survey showed better psychological well-being for women than men (Bowman, 2010), and other studies have shown no evidence of sex differences in psychological well-being (Burris et al., 2009; Lothes & Nanney, 2020). However, because these studies included samples of primarily residential students or did not report students’ housing status, it is not clear whether the findings would be similar for commuter students. Additional investigation is needed to better understand mental health and well-being among commuter college students, including whether there are differences by sex.

In sum, the unique circumstances of commuter college students present stressors that may impact their well-being, yet this is an area of research that has been largely unexplored. Thus, the purpose of the current study was to examine mental health and lifestyle health behaviors among commuter college students using psychometrically validated scales and to evaluate associations between lifestyle health behaviors and mental health in this sample of commuter college students. With this purpose, we proposed the following exploratory research questions: 1) What is the prevalence of depression, anxiety, inactivity, and poor sleep quality among commuter college students? 2) Do these prevalences differ by sex? and 3) Are there sex differences in other mental health variables (perceived stress, happiness, positive and negative mood)? Additionally, given the benefits of physical activity and sleep, we examined the association between these lifestyle health behaviors and mental health. Specifically, we hypothesized that higher levels of physical activity and better sleep quality would be associated with less depression, anxiety, negative mood, and perceived stress and higher positive mood and happiness.

## Methods

### Participants

Participants were students from a large, urban, 4-year commuter college (approximately 16,000 undergraduates) in the northeastern United States who volunteered for the study in exchange for research credit toward their course requirement. At this institution, campus housing is extremely limited (less than 300 beds) and is located ~1.5 miles (30 blocks) from campus. The vast majority of students (98%) live off-campus and commute. Recruitment was done during the Fall 2021 and Spring 2022 semesters through the college’s Sona Systems subject pool website available to students in Psychology and Management classes that have a research requirement. Inclusion criteria were being a current student aged 18 or above. There were 598 students who signed up for the study; of these, two did not consent and 10 were ineligible because they were less than 18 years old. Of the 586 eligible participants who enrolled in the study, 10 had missing data across multiple questionnaires and were excluded from analyses. Thus, the final sample was 576 student participants.

Participants were 62.3% female, 32.0% Hispanic or Latino/a, and 81.2% non-White; 94.8% were full-time students and 46.5% were employed at least part-time. Their mean age was 20.31 years (SD = 3.02) and 93% were between the ages of 18–24. See Table 1 for participant demographics.

**Table 1.** Sample demographics (*N* = 576)

Demographic variables	% (n)
Sex^a^	
Female Male	62.3 (357) 37.7 (216)
Race^a^	
American Indian or Alaska Native Asian Black/African American Native Hawaiian or Other Pacific Islander White More than one race Other	1.4 (8) 40.4 (232) 12.7 (73) 0.7 (4) 18.8 (108) 6.8 (39) 19.2 (110)
Ethnicity^a^	
Hispanic or Latino/a Not Hispanic or Latino/a	32.0 (184) 68.0 (391)
Year in school	
1st year undergraduate 2nd year undergraduate 3rd year undergraduate 4th year undergraduate 5th year undergraduate Other	34.9 (201) 23.6 (136) 24.0 (138) 14.4 (83) 2.1 (12) 1.0 (6)
Student Status	
Full-time Part-time	94.8 (546) 5.2 (30)
Employment Status	
Full-time Part-time Not employed	8.0 (46) 38.5 (222) 53.5 (308)

^a^Three participants declined to answer the sex question; two declined to answer the race question; one declined to answer the ethnicity question.

### Measures

The following self-report measures were included in the Qualtrics web survey.

#### Demographics

Demographic characteristics including age, sex, race, ethnicity, year in school, and employment status were assessed.

#### Mood

##### Positive and Negative Affect

The Positive and Negative Affect Schedule (PANAS) was used to measure mood. Specifically, the PANAS measures 10 positive (e.g., interested, excited, enthusiastic) and 10 negative (e.g., upset, irritable, nervous) affective states (Watson et al., 1988). Respondents rated the extent to which they experienced each mood state during the past few weeks on a 5-point scale from 1 (very slightly or not at all) to 5 (extremely). The positive affect (PA) and negative affect (NA) subscales are summed separately, with total PA and NA scores ranging from 10–50. The PANAS has demonstrated good reliability and validity in diverse samples, including college students, and has been used extensively to measure positive and negative affect (Crawford & Henry, 2004; Serafini et al., 2016; Watson et al., 1988). In the current study, internal consistency reliability for the PANAS PA and NA subscales, as measured by Cronbach’s alpha, was .90 and .88, respectively.

##### Depression

Depressive symptoms were measured using the 10-item Center for Epidemiologic Studies Depression scale (CESD-10 (Andresen et al., 1994); derived from the original validated 20-item measure (Radloff, 1977). Items are rated in terms of how respondents have felt over the past week using a scale of 0 (rarely or none of the time) to 3 (most or all of the time). For example, “I felt depressed” and “I felt that everything I did was an effort”. Total scores on the CESD-10 range from 0–30 with higher scores indicating greater symptom severity. A cutpoint of ≥ 10 has been identified as equivalent to a cutoff of ≥ 20 on the full-length CESD (Andresen et al., 1994) as indicating clinically significant depressive symptoms. The CESD-10 is a widely used and validated tool to screen for depression in adults, adolescents, and college students (Andresen et al., 1994, 2013; Bradley et al., 2010; González et al., 2017; Noel et al., 2023). In the present study, Cronbach’s alpha for the CESD-10 was .83.

##### Happiness

Personal happiness was measured using the shortened 8-item Oxford Happiness Questionnaire (OHQ; Hills & Argyle, 2002) which was derived from the Oxford Happiness Inventory (Argyle et al., 1989). Items on the OHQ are rated on a 6-point scale from 1 (strongly disagree) to 6 (strongly agree). For example, “I feel that life is very rewarding” and “I can fit in everything I want to”. Short form total scores range from 8–48, with higher scores reflecting greater happiness. The OHQ has good validity and reliability and has been used to measure happiness in the general population and in college student samples (Francis et al., 1998; Hills & Argyle, 2002; Jiang et al., 2022; Robbins et al., 2010). In the current study, Cronbach’s alpha for the OHQ short form was .79.

#### Anxiety and stress

##### Generalized Anxiety

The Generalized Anxiety Disorder-7 (GAD-7) is a 7-item tool used to screen for and assess severity of GAD (Spitzer et al., 2006). Respondents rated the frequency of symptoms (e.g., worrying too much about different things, trouble relaxing) over the past 2 weeks using a scale of 0 (not at all) to 3 (nearly every day). The GAD-7 ranges from 0 to 21, with a score of 10 identified as a reasonable cut-off for GAD. The GAD-7 is a valid and reliable instrument that has been widely used to screen for generalized anxiety in diverse samples, including college students (Byrd-Bredbenner et al., 2020; Plummer et al., 2016; Roberts et al., 2023; Spitzer et al., 2006). In the present study, Cronbach’s alpha for the GAD-7 was .93.

##### Perceived Stress

The 10-item Perceived Stress Scale (PSS; Cohen & Williamson, 1988; Cohen et al., 1983) was used to measure the degree to which life situations are perceived as stressful over the past month (e.g., felt unable to control important things in life, felt confident about ability to handle personal problems). Items are rated on a scale of 0 (never) to 4 (very often), with total scores ranging from 0–40 and higher scores indicating greater perceived stress. The PSS is a widely used and psychometrically validated instrument for measuring perceived stress in diverse samples of the general population and in college students (Cohen & Janicki-Deverts, 2012; Graves et al., 2021; Roberts et al., 2023). In the current study, Cronbach’s alpha for the PSS was .81.

#### Physical activity

Physical activity level over the past month was measured using the Stanford Leisure-Time Activity Categorical Item (L-Cat; Kiernan et al., 2013). The L-Cat is a single-item tool for measuring physical activity levels using six descriptive categories ranging from inactive to very active based on the 2007 American College of Sports Medicine (ACSM) guidelines which recommend that healthy adults ages 18–65 engage in moderate intensity aerobic activity on 5 or more days per week for 30 minutes or more each time (Haskell et al., 2007). The first three categories of the L-Cat reflect levels of activity below the ACSM guidelines, the fourth category is consistent with the guidelines, and the final two categories reflect activity levels that are above the guidelines. The L-Cat has demonstrated good validity and reliability when evaluated against self-reported and objectively measured physical activity (Kiernan et al., 2013; Ross et al., 2018). Participants also were asked to indicate the average number of days per week they engaged in moderate or vigorous intensity activity (e.g., brisk walking, running, swimming, riding a bike) over the last month.

#### Sleep quality

The Pittsburgh Sleep Quality Index (PSQI) was used to measure sleep quality during the past month. The PSQI is a widely used, validated 19-item self-report questionnaire that measures sleep quality across 7 components (subjective sleep quality, sleep latency, sleep duration, sleep efficiency, sleep disturbance, use of sleep medications, and daytime dysfunction; Buysse et al., 1989). Each component score ranges from 0 (no difficulty) to 3 (severe difficulty). The sum of the component scores yields a total score that ranges from 0–21, with higher scores indicating poorer sleep quality. Total scores were dichotomized into poor sleep quality (total score > 5) and good sleep quality (total score ≤ 5) consistent with recommendations (Buysse et al., 1989). The PSQI demonstrates good reliability and validity in clinical and non-clinical samples (Buysse et al., 1989; Mollayeva et al., 2016). In the current study, Cronbach’s alpha for the PSQI was .68.

### Procedure

This study was approved by the College’s Institutional Review Board prior to data collection (#2021–0526). This was a cross-sectional, anonymous, web-based survey delivered through Qualtrics. Participants accessed the survey link through the SONA web platform, completed an online consent, and proceeded through to the online survey. Participants received research credit upon survey completion.

### Data analysis

Descriptive statistics (means, standard deviations, frequencies) were calculated for demographic variables. Chi square analyses were used to evaluate differences on categorical variables and effect size was measured using the phi (ɸ) coefficient, with values of 0.1, 0.3, and 0.5 indicating small, medium, and large effects, respectively (Cohen, 1992). Sleep quality, physical activity level, depression, and anxiety were dichotomized based on relevant clinical cutoffs (poor/good sleep quality; below/at or above recommended activity levels; below/at or above threshold for depression and anxiety). Pearson correlation coefficients were conducted to evaluate bivariate associations on continuous measures of sleep, physical activity, and mental health. The adequacy of measurement models for variables that included more than one item was evaluated by estimating several confirmatory factor analytical models in *Mplus* using a maximum likelihood estimator with robust standard errors (Muthén & Muthén, 2017). Goodness of fit was evaluated using standard criteria (i.e., CFI > .90, RMSEA < .08, and SRMR < .08; Brown, 2015). However, these cutoffs are to be interpreted as reasonable guidelines, rather than inflexible golden rules (Bazzoli, 2024). Group differences on continuous mental health scale scores were evaluated using multigroup structural equation models to empirically test whether there are any sex, physical activity, and sleep quality effects on mental health indicators. These models were estimated by imposing equality constraints on like indicators and intercepts and latent factors were identified and given a metric using effects coding (Little et al., 2006). Wald chi square tests were used to establish whether latent means were different across groups and latent Cohen’s d was used as a measure of the magnitude of effects, with guidelines of 0.2, 0.5, and 0.8 for small, medium, and large effects, respectively (Cohen, 1992). Tests of statistical significance were based on alpha set at .05.

There were three participants who declined to report their sex, so those participants were excluded from analyses of sex differences. There were 39 participants who had missing or invalid data on the PSQI preventing a total score from being computed, thus those participants were excluded from analyses involving the PSQI.

## Results

### Prevalence of depression, anxiety, inactivity, and poor sleep quality and differences by sex

#### Depression & anxiety

The mean CES-D10 score in this sample was 11.38 (SD = 6.07). Overall, 58.6% of students screened positive for depression based on a CES-D10 cut-off score of 10, with significantly more females than males meeting this threshold (65.2% v. 47.7%, *p* < .001, ɸ=.17). The mean GAD-7 score was 8.04 (SD = 5.97); 37.8% of students screened positive for anxiety with a cut-off score of 10, and significantly more females met this threshold than males (44.3% v. 27.3%, *p* < .001, ɸ=.17).

#### Physical activity & sleep quality

Participants reported an average of 2.42 ± 1.91 days/week of moderate or vigorous intensity activity in the last month. Less than one quarter (23.3%) of participants met or exceeded recommended levels of physical activity according to ACSM guidelines (≥30 minutes of moderate intensity activity on ≥5 days per week). Significantly fewer females than males reported levels of physical activity at or above the guidelines (15.4% v. 36.1%, *p* < .001, ɸ=.24).

The mean PSQI total score was 6.53 (SD = 3.38). Overall, 57.4% of the sample was classified as having poor sleep quality using the threshold total score > 5 on the PSQI. Significantly more females than males met the threshold for poor sleep quality (61.1% v. 51.0%, *p* < .05, ɸ=.10).

### Measurement models for associations between sex, physical activity, sleep quality, and mental health

Before estimating group differences to evaluate the associations between physical activity, sleep quality, and mental health, we ensured that the measurement model for each of the constructs fit the data adequately. The positive PANAS dimension showed good fit to the data, as it met conventional fit criteria (χ^2^(35) = 193.31, CFI = .93, RMSEA = .08, SRMR = .04), showed substantial and significantly different from zero factor loadings, and minimal residuals. The negative dimension did not initially fit the data acceptably, and we respecified a model with two correlated residuals. These residuals, items 7 (scared) and 20 (afraid) and items 6 (guilty) and 13 (ashamed) are semantically very close, and it is reasonable to assume that these items may share some systematic variance. The respecified model fit the data well, as it met conventional fit cutoffs (χ^2^(33) = 187.16, CFI = .92, RMSEA = .08, SRMR = .04), showed substantial and significantly different from zero factor loadings, and minimal residuals.

The happiness measurement model showed acceptable fit to the data, as it met conventional fit (χ^2^(20) = 101.30, CFI = .90, RMSEA = .08, SRMR = .05), showed substantial and significantly different from zero factor loadings, and minimal residuals.

The depression measurement model did not show acceptable fit initially, and we respecified the model by adding correlated residuals between item 5 (I was happy) and 8 (I felt hopeful about the future), both of which are reverse coded and measure happiness. The respecified model fit the data well, as it met standard fit cutoffs (χ^2^(34) = 90.26, CFI = .96, RMSEA = .05, SRMR = .04), showed substantial and significantly different from zero factor loadings, and minimal residuals.

The perceived stress measurement model estimated two latent factors to represent perceived helplessness and perceived self-efficacy but did not fit the data well initially. We respecified it by adding a correlated residual between items 9 (how often have you been angered because of things out of your control) and 10 (how often have you felt difficulties were piling up so high you could not overcome them), due to their close semantic proximity, and it fit the data well, as it met standard fit cutoffs (χ^2^(33) = 316.21, CFI = .90, RMSEA = .08, SRMR = .06), showed substantial and significantly different from zero factor loadings, and minimal residuals.

The anxiety measurement model fit the data well, as it met standard fit cutoffs (χ^2^(14) = 87.54, CFI = .96, RMSEA = .08, SRMR = .03), showed substantial and significantly different from zero factor loadings, and minimal residuals.

### Associations between sex, physical activity, sleep quality, and mental health

Estimated latent means, latent mean differences, Wald tests, and latent Cohen’s d are shown in Table 2; these means reflect the optimally weighted average of all manifest indicators’ means loading onto a latent factor (see Chapter 3 in Little (2013) for more details on estimation) and should not be interpreted as or compared to raw means obtained using other estimation procedures. For clinical reference, raw mental health scale means by sex, physical activity, and sleep quality are presented in Table 3. We found differences by sex across most mental health indicators (happiness latent means were not significantly different): males showed significantly higher levels of positive affect and significantly lower levels of negative affect, anxiety, depression, and perceived stress compared to females. Consistent with our hypotheses, physical activity and sleep were positively associated with better mental health. Specifically, we found significant differences on all indicators of mental health between groups based on physical activity: students who met/exceeded ACSM recommendations for physical activity reported significantly higher levels of positive affect and happiness, and significantly lower levels of negative affect, anxiety, depression, and perceived stress compared to students who did not. Similarly, we found significant differences on all indicators of mental health between groups based on sleep quality: students who had good sleep quality reported significantly higher levels of positive affect and happiness, and significantly lower levels of negative affect, anxiety, depression, and perceived stress compared to students who had poor sleep quality.

**Table 2.** Differences in mental health as a function of sex, physical activity, and sleep quality

	Group 0 Mean Estimate	Group 1 Mean Estimate	Difference	Wald test	p-value	Latent Cohen’s d
*Sex (0 = males 1 = females)*						
Positive Affect	3.12	2.95	0.17	5.66	.02	0.22
Negative Affect	2.19	2.69	0.50	57.24	<.001	0.71
Depression	1.03	1.17	0.14	11.94	<.001	0.34
Anxiety	0.80	0.99	0.19	11.79	<.001	0.33
Stress	1.34	1.80	0.46	43.38	<.001	0.64
Happiness	4.32	4.22	0.10	1.92	.17	–
*Physical Activity (0 = inactive 1 = active)*						
Positive Affect	2.92	3.32	0.40	22.30	<.001	0.54
Negative Affect	2.54	2.23	0.31	17.71	<.001	0.41
Depression	1.15	0.98	0.17	16.36	<.001	0.44
Anxiety	0.98	0.74	0.24	16.49	<.001	0.40
Stress	1.71	1.38	0.33	17.01	<.001	0.45
Happiness	4.16	4.59	0.43	28.13	<.001	0.57
*Sleep Quality (0 = poor 1 = good)*						
Positive Affect	2.90	3.17	0.27	15.99	<.001	0.36
Negative Affect	2.69	2.19	0.50	57.24	<.001	0.71
Depression	1.33	0.79	0.54	275.33	<.001	1.77
Anxiety	1.14	0.63	0.51	96.19	<.001	0.91
Stress	1.94	1.23	0.71	126.20	<.001	1.10
Happiness	4.06	4.57	0.51	48.73	<.001	0.70

Mean differences and latent Cohen’s d are in absolute values for ease of interpretation and all Wald’s chi square tests have 1 df. Positive and Negative Affect: PANAS; Depression: CES-D10; Anxiety: GAD-7; Perceived Stress: PSS; Happiness: OHQ-8.

**Table 3.** Group means on mental health scales

	Group 0 Mean (SD)	Group 1 Mean (SD)
*Sex (0 = males 1 = females)*		
Positive Affect	31.18 (8.68)	29.42 (7.51)
Negative Affect	22.79 (7.87)	25.75 (8.01)
Depression	10.24 (5.93)	12.10 (6.04)
Anxiety	6.41 (5.63)	9.03 (5.97)
Perceived Stress	17.94 (6.61)	20.68 (6.48)
Happiness	31.68 (7.38)	30.83 (6.84)
*Physical Activity (0 = inactive 1 = active)*		
Positive Affect	29.09 (7.53)	33.36 (8.67)
Negative Affect	25.35 (8.19)	22.27 (7.27)
Depression	11.96 (6.11)	9.55 (5.53)
Anxiety	8.44 (6.02)	6.74 (5.66)
Perceived Stress	20.28 (6.49)	17.57 (6.80)
Happiness	30.41 (6.86)	33.61 (7.14)
*Sleep Quality (0 = poor 1 = good)*		
Positive Affect	29.05 (8.14)	31.52 (7.31)
Negative Affect	27.00 (7.81)	21.79 (7.21)
Depression	13.79 (5.66)	8.17 (4.96)
Anxiety	10.15 (5.75)	5.27 (4.86)
Perceived Stress	22.10 (6.07)	16.44 (6.04)
Happiness	29.30 (6.49)	33.44 (7.10)

Positive and Negative Affect: PANAS; Depression: CES-D10; Anxiety: GAD-7; Perceived Stress: PSS; Happiness: OHQ-8.

We also examined the associations between physical activity and sleep quality with mental health indicators in a continuous fashion (i.e., without grouping based on clinically relevant cutoff scores for physical activity and sleep). Table 4 reports the zero-order correlations among study variables. Correlation patterns mirror the results of the multigroup structural equation models. Specifically higher levels of physical activity and better sleep quality were associated with better mental health indicators (lower depression, anxiety, negative mood, and stress, and higher positive mood and happiness).

**Table 4.** Correlations for physical activity, sleep quality, and mental health

	1	2	3	4	5	6	7
1. Physical Activity	–						
2. Sleep Quality	−.13***	–					
3. Positive Affect	.28***	−.22***	–				
4. Negative Affect	−.22***	.35***	−.16***	–			
5. Depression	−.24***	.57***	−.45***	.61***	–		
6. Anxiety	−.20***	.48***	−.23***	.67***	.70***	–	
7. Stress	−.24***	.53***	−.43***	.60***	.77***	.67***	–
8. Happiness	.27***	−.40***	.59***	−.46***	−.66***	−.47***	−.64***

Positive and Negative Affect: PANAS; Depression: CES-D10; Anxiety: GAD-7; Perceived Stress: PSS; Happiness: OHQ-8. ***p<.001.

## Discussion

This study reports on findings from a cross-sectional web-based survey of mental health and lifestyle health behaviors in a large sample of racially and ethnically diverse undergraduates attending an urban, public commuter college. The results show high rates of depression, anxiety, and poor sleep quality and low rates of engagement in regular physical activity in the sample overall. These results address a gap in the literature on college student well-being in that they identify mental health and lifestyle health behavior problems among commuter college students. We found that almost 60% of students screened positive for depression based on the CES-D10. This is higher than the 27% lifetime diagnosis of depression reported in the 2024 ACHA data (ACHA, 2024) and the 2022–2023 Healthy Minds Study National Data Report showing that 41% of college students met thresholds for depression based on the PHQ-9 (Eisenberg et al., 2023). While the CES-D10 is a screening measure that identifies depression risk and not a diagnostic tool, the high rate of depression symptoms in this sample of commuter students is alarming. Our findings show that 38% of students screened positive for generalized anxiety based on the GAD-7. This is in line with results of the 2022–2023 Healthy Minds Study in which 36% of students met GAD-7 threshold for anxiety (Eisenberg et al., 2023), and the 2024 ACHA data, in which 35% of students had a lifetime anxiety disorder diagnosis (ACHA, 2024).

There is scant research on the mental health of commuter college students, yet this is an important subgroup to study because their college experience differs from that of their residential college peers. Beyond the unique stressors they face related to balancing competing life roles while attending college, commuter students are managing time and transportation constraints and are less likely to access campus support that may mitigate the effects of stress. Commuter college students tend to spend less time on campus outside of classes when they might utilize campus-based resources such as the counseling center, fitness center, or academic support services and they report a lower sense of institutional belonging compared to residential students (Alfano & Eduljee, 2013; Jacoby & Garland, 2004; Newbold et al., 2011). These findings are noteworthy given that students’ sense of institutional belonging predicts depressive symptoms, college persistence, and student engagement (Dutcher et al., 2022; Gillen O’Neel, 2021; Gopalan & Brady, 2020; Gopalan et al., 2022). Additional research is needed to better understand institutional belonging in commuter college students and the degree to which these and other factors related to the commuter experience may impact student mental health and use of campus support resources. Such research can be used to inform institutional outreach efforts to better address the needs of commuter students.

Over 57% of students in the current study were classified as having poor sleep quality, which is in line with the college student literature (Becker et al., 2018). Moreover, poor sleep quality was strongly associated with lower overall psychological well-being (i.e., higher negative affect, depression, anxiety, and perceived stress and lower positive affect and happiness). Effect sizes for depression, anxiety and perceived stress were large (>.80) although this may be inflated for the association between sleep and depression given that sleep disturbance is a symptom of depression and assessed by the CES-D10. Nonetheless, the magnitude of these effects points to the clinical implications for sleep on mental health and is consistent with prior studies demonstrating the negative impact of sleep deprivation on mental health (Hershner & Chervin, 2014; Orzech et al., 2011). While some factors that interfere with sleep quality of commuter students may overlap with those of residential students (e.g., time spent on devices) others are likely unique to their commuter circumstances (e.g., time spent commuting to/from school and work). Despite the prevalence of sleep disturbance among college students, research shows that few seek help to manage their sleep (Qin & Brown, 2017). Taken together, these findings raise concern about college students’ sleep health and highlight opportunities for campus wellness initiatives that both educate students about the negative effects of insufficient sleep and promote strategies for good sleep hygiene such as maintaining a regular nighttime sleep schedule and limiting use of blue light emitting devices (e.g., laptops, smartphones, tablets) before bed (American Academy of Sleep Medicine, n.d.). Novel approaches or modalities that accommodate commuter students’ limited time on campus such as digital interventions that leverage social media platforms or text-messaging could be explored to reduce barriers and optimize access.

In the current study, participation in regular physical activity was low, with only 23% of students engaging in the recommended 150 minutes of moderate-intensity physical activity per week. This is considerably lower than the rates of physical activity among college students in the 2024 ACHA data, which show that 43% meet physical activity recommendations (ACHA, 2024). This is one study conducted on a single urban commuter campus, and therefore we interpret these results with caution. Additional research assessing engagement in physical activity among students at other commuter institutions and/or directly comparing activity levels of residential and commuter college students is needed to determine whether physical activity is significantly lower among undergraduates who commute. While beyond the scope of the present study, further examination of barriers related to physical activity among commuter college students is warranted. Students who commute may face distinct challenges to being active compared to students living in dorms on residential campuses. For example, they may find it more difficult to use university fitness centers because of scheduling constraints that limit their time on campus and practical issues such as access to shower facilities or lockers to store belongings. Prior studies have shown that living off campus and distance from exercise facilities are related to lower levels of physical activity among college students (Ajibade, 2011; Kapinos & Yakusheva, 2011; Small et al., 2013). Research that explores ways to identify and effectively reduce barriers to physical activity for commuter college students would be a valuable contribution to the literature.

Notably, students in the current study who met or exceeded physical activity recommendations reported significantly more positive affect and happiness, and less negative affect, depression, anxiety and perceived stress compared to those who did not meet activity recommendations, with small to medium effect sizes. These findings are consistent with prior research showing positive associations between physical activity and psychological well-being in college students (Elliot et al., 2012; VanKim & Nelson, 2013). Moreover, a recent systematic review of physical activity intervention trials conducted with college students showed positive effects of moderate intensity activity interventions on mental health (Donnelly et al., 2024). Thus, there is considerable evidence for the benefits of establishing physical activity habits during the college years. The low rate of physical activity in the present study suggests that commuter students may need additional support to participate in regular activity. Exploring ways for commuter institutions to encourage students’ physical activity could include offering alternatives like a credit-bearing physical activity elective or campus-sponsored virtual fitness campaigns through school social media channels, which may not only facilitate student engagement in physical activity but also promote a sense of campus community.

Our findings show while there were high rates of depression, anxiety, poor sleep quality, and inactivity in the sample overall, women fared worse than men in both mental health and lifestyle health behaviors. This is consistent with research by Glavin et al. (2022) using a largely residential sample of college students and a recent study of commuter college students by Parker et al. (2023). Research points to several possible factors that may contribute to poorer well-being among female students, including dating violence (Amar & Gennaro, 2005), body dissatisfaction (Beiter et al., 2015), and lack of social support (Parker et al., 2023). In addition, commuter students face stressors of role strain and commuting in the context of feeling disconnected from campus life. Research that explores sex differences in other samples of commuter college students is warranted, however, these findings suggest there may be a specific need for more self-care and/or mental health support for commuter college females. Such initiatives may include programs to promote adaptive coping skills (Bettis et al., 2017) resiliency (Houston et al., 2017; Seppälä et al., 2020) and/or peer connection (Pointon-Haas et al., 2023). Understanding how campus communities can best strengthen the well-being of their female students is an important priority.

To meet the growing demand for mental health services, many college counseling centers have developed offerings beyond traditional individual or group counseling such as crisis intervention and telemental health (Abrams, 2022; Mitchell, 2023). Future research that explores intervention modalities to accommodate commuter students would be a valuable addition to the literature. For example, self-guided online mental health interventions (Davis et al., 2024) or stepped care models (Cornish et al., 2017) could increase commuter student access to services, and use of college social media channels or learning management systems to communicate mental health services may be helpful alternatives for commuters who spend less time on campus. In addition, given the mental health benefits of institutional belonging and interpersonal support, especially from friends (Lee & Goldstein, 2016; Pittman & Richmond, 2008; Rayle & Chung, 2008), developing initiatives that build these elements on commuter campuses could be valuable areas of focus. To optimize participation, these initiatives would need to consider practical aspects relevant to a commuter student body such as scheduling events around class times, providing student-centered places to gather, and/or offering virtual programs.

The current findings offer insight on the mental health and lifestyle health behaviors of commuter college students. This is a target population which has received little research attention and therefore this study addresses an important gap in the college student literature. Strengths of this study are that it included a large sample of racially and ethnically diverse undergraduates thereby extending the generalizability of research on college student well-being, and that it used psychometrically validated scales to assess mental health and lifestyle health behaviors which support measurement accuracy and interpretation of constructs across studies.

There are limitations of this study that must be considered. While this sample was diverse, it was recruited using a convenience sampling approach and not representative of the broader student body at this commuter institution. Moreover, the category of commuter students is heterogeneous and includes traditional-aged students attending commuter colleges or commuting to residential colleges, part-time students, and nontraditional aged college students (Jacoby, 1989). This study surveyed traditional-aged, full-time students attending an urban commuter institution. Research that captures differences in mental health and well-being across different groups of commuter students could help inform campus-based support programs. Since this was a cross-sectional survey study, the relationships presented are associations and causal inferences among variables cannot be made. The order of questionnaires was not randomized across participants thus it is possible that there were carryover effects such that questionnaires completed first influenced responses on questionnaires completed later. Finally, in this study, the PSQI had a Cronbach’s alpha of .68 which is below the generally accepted threshold of .70 (Tavakol & Dennick, 2011). While many studies, including a systematic review and meta-analysis of the PSQI (Mollayeva et al., 2016), have demonstrated Cronbach’s alpha values above .70, Becker et al. (2018) used the PSQI in a large multi-university sample of college students and reported a Cronbach’s alpha of .69. Additional research testing the internal consistency of the PSQI in college samples may be needed.

In conclusion, the results of this study reflect high rates of depression, anxiety, and poor sleep quality, and low engagement in physical activity in a diverse sample of commuter college students, particularly among women. These findings highlight opportunities for commuter institutions to address student mental health and wellness needs.

## Data Availability

The participants of this study did not give written consent for their data to be shared publicly, thus supporting data are not available.
